# The Influence of Electro-Conductive Compression Knits Wearing Conditions on Heating Characteristics

**DOI:** 10.3390/ma14226780

**Published:** 2021-11-10

**Authors:** Md. Reazuddin Repon, Ginta Laureckiene, Daiva Mikucioniene

**Affiliations:** Department of Production Engineering, Faculty of Mechanical Engineering and Design, Kaunas University of Technology, Studentų 56, LT-51424 Kaunas, Lithuania; ginta.laureckiene@ktu.lt (G.L.); daiva.mikucioniene@ktu.lt (D.M.)

**Keywords:** conductive textiles, heat generation, orthopaedic support, compression, washing, stretch

## Abstract

Textile-based heaters have opened new opportunities for next-generation smart heating devices. This experiment presents electrically conductive textiles for heat generation in orthopaedic compression supports. The main goal was to investigate the influence of frequent washing and stretching on heat generation durability of constructed compression knitted structures. The silver coated polyamide yarns were used to knit a half-Milano rib structure containing elastomeric inlay-yarn. Dimensional stability of the knitted fabric and morphological changes of the silver coated electro-conductive yarns were investigated during every wash cycle. The results revealed that temperature becomes stable within two minutes for all investigated fabrics. The heat generation was found to be dependent on the stretching, mostly due to the changing surface area; and it should be considered during the development of heated compression knits. Washing negatively influences the heat-generating capacity on the fabric due to the surface damage caused by the mechanical and chemical interaction during washing. The higher number of silver-coated filaments in the electro-conductive yarn and the knitted structure, protecting the electro-conductive yarn from mechanical abrasion, may ensure higher durability of heating characteristics.

## 1. Introduction

Smart textiles are fabrics that interact intelligently with the nearby surroundings or the consumer. The electronic textile (e-textile) is capable of sensing environmental circumstances or stimuli and providing information that can effectively respond to and adapt actions. Electrically conductive textile is a piece of smart textile that incorporates conductive fibers, yarns, fabrics, and ultimate products. Heating applications, particularly in the medical field, are a significant use of electrically conductive materials [1,2,3,4].

Knitted orthopaedic supports are designed to provide skeletal support for improving the life quality of patients. Unlike traditional knitted garments, compression garments are compact knitted garments customized for medical purposes. Compression therapy is usually applied for bandaging, compression stockings and orthopaedic supports. Compression products can be categorized into three classes according to the compression level—preventive supports, functional supports and postoperative/rehabilitation supports. They are used as knee, wrist, ankle, shoulder, elbow, or back braces and supports [5]. The orthopaedic supports are useful to those who are aged, pregnant, neonates, and suffering from motor impairment or paraplegic by improving the body anatomically and strengthening motor skills [6]. Knitted orthopaedic supports usually are produced as elastic knitted structures with supplementary silicone or metallic elements, such as straps, fasteners, hinges used for different purposes. Some of these components work together to generate a function that is relevant to the patient’s health and healing process, some of them increase wearing comfort, but all of them may change the elasticity and compression of the entire product. The rigid parts can not only notably affect compression generated by the support, but also even alter the compression class of the product [7].

Many research works are focused on orthopaedic supports and analysis of their construction, structure, properties, and behaviour during wearing [7,8,9,10,11,12,13]. Stretchable knitted structures are used for compression garments that contain elastomeric yarns and gain appropriate compression [7]. There is a strong correlation between the mechanical characteristics of the fabric and its generated pressure. It is well recognized that mechanical properties of a knitted fabric are related to the geometry of the knitted elements, knitted pattern, properties of the yarns used for knitting, and direction in which elastomeric yarns are laid in the fabric structure as well as the direction in which the fabric is used [14,15]. To generate compression and to achieve better performance of compression support, extra inlay-yarns are inserted into the construction of a knit as inlaid, floated, or plated yarns. The higher amount of compression is mainly accomplished by increasing the thickness of the elastic core of the inlay-yarn, although adjustments may also be made to the ground yarn [16,17]. It is important to notice that a different geometry of knitted structure generates different mechanical properties that are closely linked to the fabric structure, yarn properties. Compression of an orthopaedic support rely on the support area, shape, and characteristics of knitting, such as knitting pattern, density, etc. [12,17].

Heat therapy is used for relaxing the stiffness of joints and reducing pain. External heating can affect high-temperature-sensitive receptors in nerve endings, thereby activating blood flow and increasing muscle plasticity [18,19]. Thus, heated orthopaedic products will help to solve problems of low physical activity and related injuries. One of the fast-growing textile fabrics groups is wearable electronics, which significantly increases the functionality of textile products. The development of e-textiles enables the development of heated, temperature-controlled orthopaedic products that help to solve problems of low physical activity caused by chronic pain. Knitted fabrics with inserted electrically conductive fibres and yarns can be used as electro-conductive products that can generate the heat necessary for the heat therapy of compression orthopaedic supports [20,21,22,23,24,25,26,27,28]. Nevertheless, there is still a lack of information in the literature on studies of compression products with integrated heating, on changes in heating properties during wear. Around 40–45 °C temperature is required for the therapy, relaxing the stiffness of joints and reducing pain, and it is important to keep the temperature constant for a certain time. Moreover, it is necessary to solve the heating of the orthopaedic supports without adding external rigid elements that make changes in the support compression.

Orthopaedic compression products usually are indicated for the long-lasting term of wearing. It is known that conductive coating may be removed from yarns in knitted compression products during several washing, therefore a decrease of generated temperature may appear during the time of wear. Hence, the main goal of this study was to develop compression knitted specimens with incorporated electro-conductive silver coated polyamide yarns, as well as to explore heat generation characteristics and temperature changes under extension, which is essential for compression generation, and repeated washing. The tensile force and compression variations during the stress relaxation of compression knitted fabrics with various knitting structures also were investigated. The measurements were taken over a particular time (10 min) to see when the heating action stabilized.

## 2. Experimental Design

**Figure 1:**
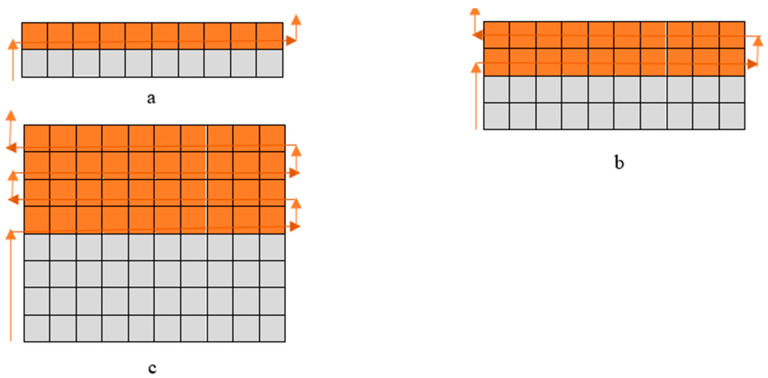
Images and principle scheme of conductive yarn layout on the technical back side of a specimen (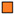
—courses with conductive yarn, 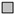
—courses without conductive yarn, 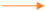
—path of conductive yarn): (**a**) EFL1 and EFH1; (**b**) EFL2 and EFH2; (**c**) EFL3 and EFH3 [29].

The ELITEX 66tex/f12_PA/Ag (silver coated polyamide) and 235tex/f34_PA/Ag yarns were used to fabricate specimens designed for orthopaedic compression support using a flat double needle-bed 14E gauge knitting machine. The knitting structure and amount of conductive yarn used in the pattern are presented in Table 1. In order to position conductive yarns on one (close to the skin) surface, the half-Milano rib structure was selected; consequently, conductive yarns were utilized only in the pattern’s single jersey courses. In addition, the conductive yarn was plated with PA6.6 yarn to cover and protect it from mechanical abrasion. Figure 1 presents the principal arrangements of the conductive yarn outline in the knitted pattern. In order to develop the stretchable structure able to generate compression, the elastomeric inlay-yarns were inserted into every rib course of the combined half-Milano structure. To develop the rib courses, the combination of 7.8 tex ×4 PA6.6 and 4.4 tex PU (polyurethane), double covered by 4.4 tex PA6.6, yarns were employed as the ground yarn. PA/Ag yarns of 66 tex (in EFL group) and 235 tex (in EFH group) were applied in plated single jersey courses as the ground yarns, while 7.8 tex ×4 PA6.6 yarn was utilized in these courses as the plating yarn. For elastomeric inlay yarn, 114.5 tex PU, double covered by 7.8 tex ×4 PA6.6, was utilized and laid in every second course of the pattern. Table 2 highlights the key characteristics of electro-conductive knitted samples.

**Figure 2:**
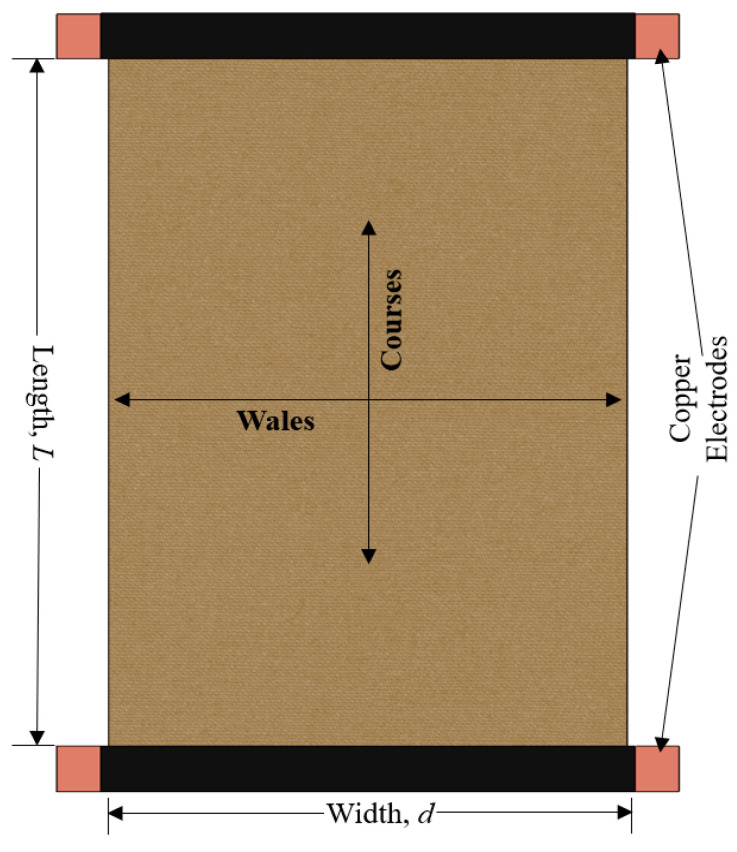
Arrangement of fabric for resistance assessment.

**Figure 3:**
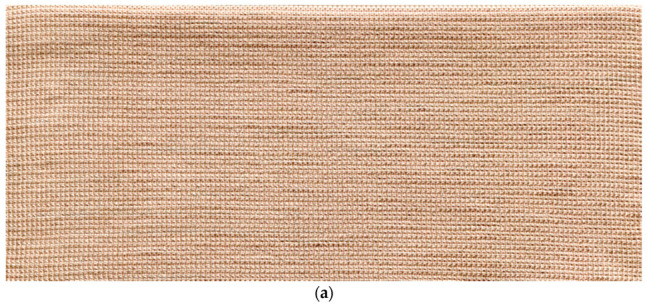

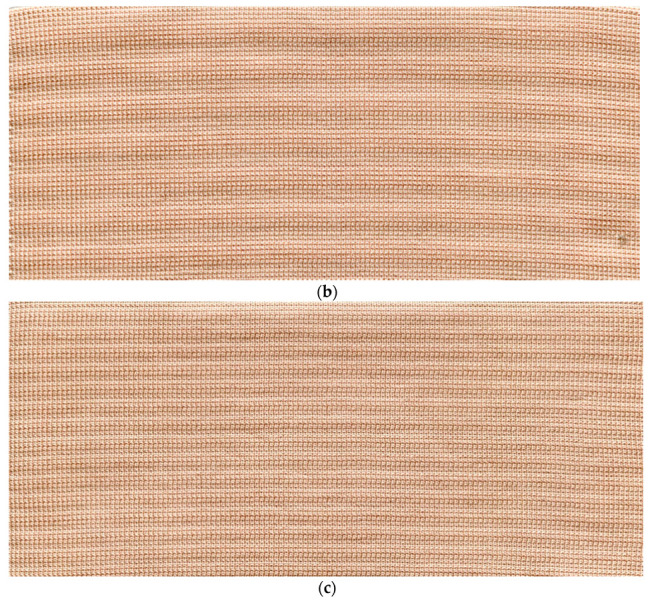
Images of conductive fabrics: (**a**) EFL1 and EFH1; (**b**) EFL2 and EFH2; (**c**) EFL3 and EFH3.

The surface temperature was measured by the infrared thermometer. The FLIR InfraCam SD thermal imager was used for thermal imaging of the samples. Thermal imager specifications of this device are identified as temperature measurement range: −10 °C to +350 °C; spectral range: 7.5 to 13 µm; accuracy: ±2%; image frequency: 9 Hz and detector type: focal plane array. The resistance of the knitted samples was assessed by using MASTECH MY 68-Digital Multimeter. Two copper plates of identical dimensions were fixed on the contrary ends of the sample and connected with the DC power source to measure the current of the corresponding applied voltage (Figure 2). The images of designed electro-conductive fabrics are given in Figure 3.

Universal testing machine ZWICK/Z005 was used to investigate compression properties followed by Standard EN ISO 13934-1:2000. Stretching to course direction was chosen because compression supports are stretched in the transversal orientation to produce compression while wearing. The tensile speed was 100 mm/min, the distance between clamps was 150 cm, pretension 2 N, sensor 5 kN. Samples were stretched to the fixed elongation of 10%, 20%, 30%, 40%, and 50% and held for the selected period in this position. The stress was recorded as a function of the time. The testing machine was operated by the testXpert^®^ software. The Laplace formula was used to determine the compression of the designed samples:

(1) $$P=\frac{2\cdot \pi \cdot F}{S}$$

where *P* is the pressure in Pa, *F* is the tensile force in *N*, *S* is the area of the specimen in m^2^.

Temperature changes on the fabric surface were evaluated during the 600 s period. The temperature was recorded each 10 s during the first minute of observation, and then each 20 s starting from the first minute to the end of the research. Average values were calculated from four elementary measurements.

The washing test was done according to standard test method EN ISO 6330:2012, using SIEMENS varioPerfect iQ700 washing machine. Washing characteristics: water temperature 40 °C, washing time 15 min, rinse time 3 min, spin time 2 min. The shrinkage of the laundered specimens in longitudinal and transverse directions was measured after each washing and drying cycle. The shrinkage values were calculated according to the formula:

(2) $$\lambda =\frac{L-L_{0}}{L_{0}}\times 100\%$$

where *L*_0_ is the initial fabric dimension in mm (before washing and drying); *L* is the fabric dimension in mm after washing and drying. In different sections of the samples, measurements were repeated 20 times and the average was recorded.

All experiments were carried out in a standard atmosphere (20 ± 2 °C temperature and 65 ± 4% humidity) in compliance with Standard LST EN ISO 139:2005.

## 3. Results and Discussion

### 3.1. Voltage (V) Optimization for Target Temperature (T)

**Figure 4:**
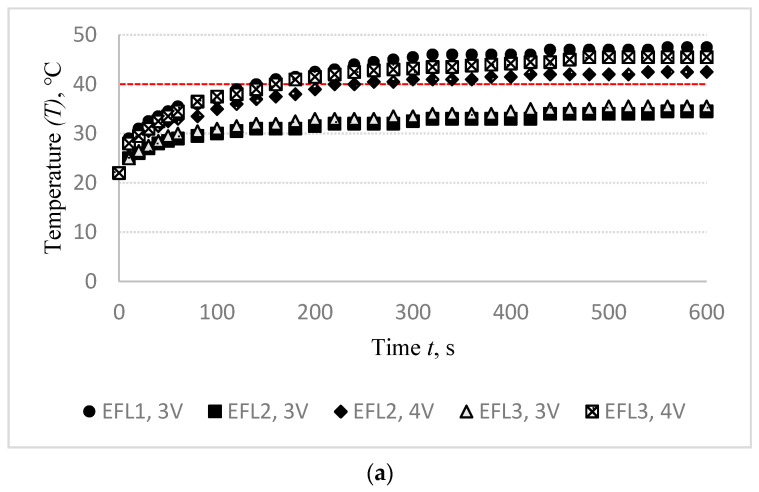

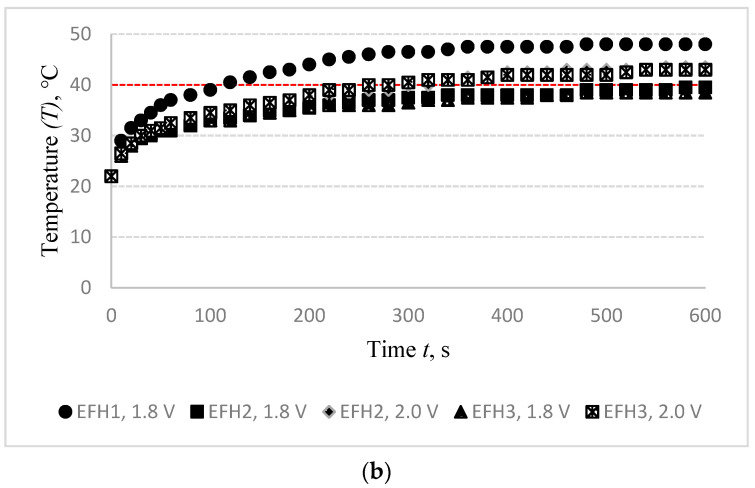
Target temperature observation of designed specimens of EFL group (**a**) and EFH group (**b**) during the 600 s period by applying different voltages.

In order to ensure the positive influence of heating on the healing process, the target temperature for orthopaedic heated supports should be not less than 40 °C. DC power source was connected to heat up the electro-conductive fabrics. Heat generation and applied voltage are exponentially reliant. The fabric surface temperature was recorded at the fixed voltage in 10 s intervals during the first minute of measurement, and every 20 s from the first minute until the end of the test, i.e., till 10 min (600 s). Changes in the surface temperature of EFL1, EFL2, EFL3, EFH1, EFH2, and EFH3 structured electro-conductive fabrics during the 600 s period by applying different voltages are shown in Figure 4.

The observed outcomes exhibit the time-dependent dynamics of the temperature variations on the surface of specimens. The voltage required to reach the targeted temperature strongly depends on both the linear density of the conductive yarn and the density of the conductive rows in the knitted structure. It was found that the 40 °C temperature of the EFL1 specimen was reached by applying a 3.0 V energy source, while 4.0 V voltage was needed for EFL2 and EFL3 to reach this temperature. For EFH group samples, 1.8 V voltage was enough to reach the target temperature for EFH1, while 2.0 V voltage was needed for EFH2 and EFH3 to reach the required temperature. The higher voltage applied creates more current and it generates more energy which is released as heat and raises the temperature in the electro-conductive fabric surface. EFL group knits exhibit higher resistance than the EFH group because of the lower linear density of the conductive yarn. By applying a voltage of 3 V, the target temperature was reached by the EFL1 variant but not reached by EFL2 and EFL3. The 4.0 V voltage was applied for EFL2 and EFL3 to reach the target temperature because approximately the twice lower amount of conductive yarn was used in the knitting pattern of these specimens. The same scenario was observed for the EFH group, too. The target temperature on the surface of the EFH1 variant was reached by applying 1.8 V voltage, while for EFH2 and EFH3 variants 2.0 V voltage was needed to reach the target temperature.

**Figure 5:**
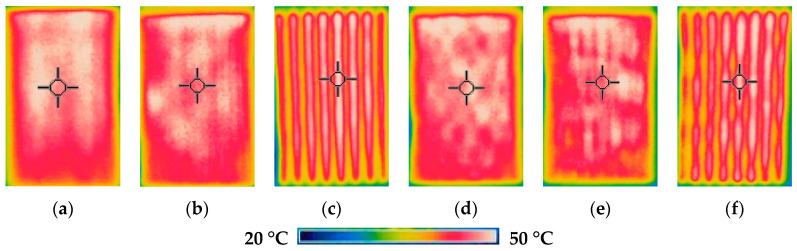
Thermal images of EFL1 (**a**), EFL2 (**b**), EFL3 (**c**), EFH1 (**d**), EFH2 (**e**), and EFH3 (**f**) structured electro-conductive fabrics after 10 min of constant voltage application.

Thermal images of the heated fabrics surface by using the set voltage (3.0 V for EFL1, 4.0 V for EFL2 and EFL3, 1.8 V for EFH1, 2.0 V for EFH2 and EFH3) after 10 min observation are presented in Figure 5.

Figure 4 highlights the impact of knitting pattern, i.e., quantity as well as distribution of the conductive yarn in the knitting pattern, where red colour denotes the highest temperature and blue colour, accordingly, the lowest temperature. Visually, thermal images indicate that despite the reached targeted overall surface temperature, the structures EFL3 and EFH3 demonstrate high unevenness of temperature on their surface. This unevenness appeared due to the different distribution of the conductive yarn in the knitting pattern (see in Table 1), i.e., due to the higher distance between courses with and without the conductive yarn compared to EFL1, EFL2, EFH1, and EFH2. The experimental findings also revealed the temperature difference between the edges and the middle of the heating area. This occurred because of high loss of heat through radiation and air convection in the sides correlated to the middle zone.

### 3.2. Effect of Elongation (ε) on Temperature (T) Characteristics

Compression supports are elastic products containing elastomeric yarns with an engineered compression gradient that can be worn on limbs, upper, lower, or full body to use for compression therapy. Elastomeric inlay-yarns are used in the knitted structure to give the most effective compression generation. Heated orthopaedic support, however, requires compression. Therefore, the conductive yarns are bent into the loop rather than straight laid yarns in this arrangement. This allows for support extension at the required level without causing severe damage to the electro-conductive yarn.

The samples were stretched to 10%, 20%, 30%, 40%, and 50% at fixed elongation in order to imitate the possible wear conditions and to find what influence it has on the temperature generation. The compression values obtained at the different stretch levels of investigated fabrics are exhibited in Table 3.

**Figure 6:**
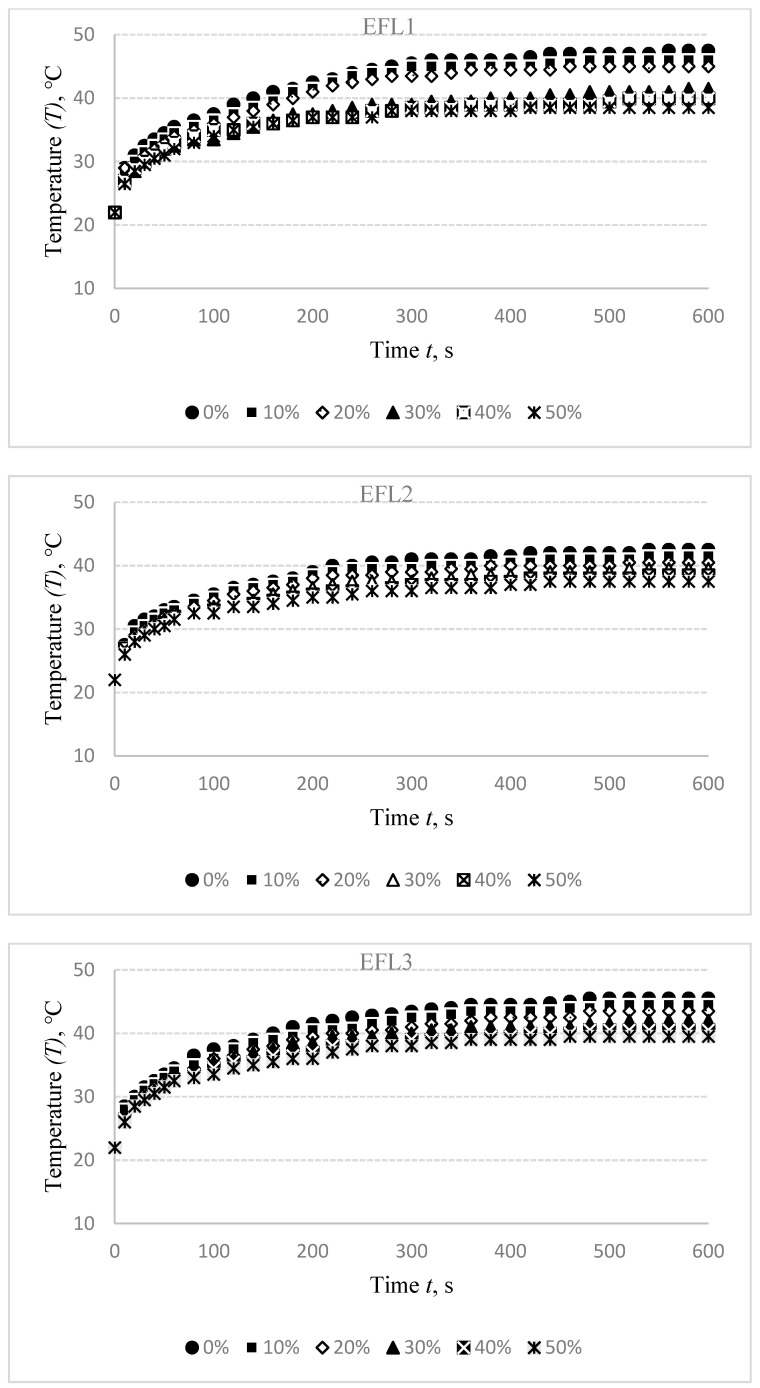

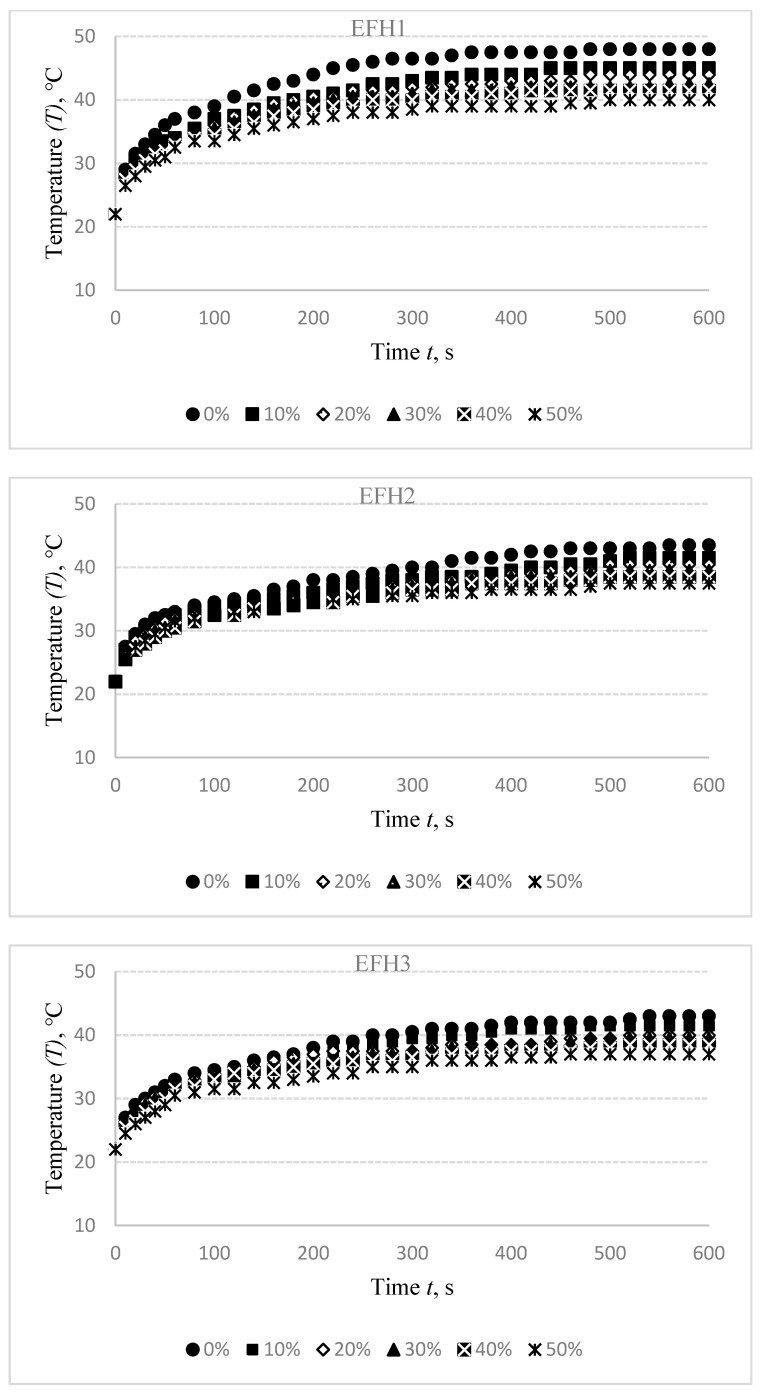
Temperature changes of EFL1, EFL2, EFL3, EFH1, EFH2, and EFH3 structured electro-conductive fabrics during the 600 s period at different stretch levels.

As it was set in the first part of the experiment, 3.0 V voltage was applied for the EFL1 specimen, 4.0 V for EFL2 and EFL3, 1.8 V for EFH1, 2.0 V for EFH2 and EFH3 to reach the targeted temperature. The experimental results are presented in Figure 6.

The stretch of the compressive electro-conductive knitted fabric has a significant negative influence on the heat generation, as shown in Figure 6; however, the heat generation dynamics during the time have a similar character in both non-stretched and stretched states. The difference in temperature between non-stretched and 10% stretched states after 600 s was determined to be 1.5 °C for EFL1, 1 °C for EFL2 and EFL3, and this difference increases the stretch level. In 20% stretch state it is, accordingly, 2.5 °C and 2 °C; in 30% state—6 °C for EFL1, 3 °C for EFL2, and 3.5 °C for EFL3; in 40% state—accordingly, 7.5 °C, 4 °C, and 5 °C; and the difference in 50% state is, accordingly, 9 °C, 5 °C, and 6 °C. This difference is also high in EFH group arrangements. Comparing temperatures in non-stretched and 10% stretched state, the difference is 3 °C for EFH1, 2 °C for EFH2, and 1.5 °C for EFH3; in 20% state it is, accordingly, 4 °C, 3 °C, and 3 °C; in 30% state—accordingly, 5.5 °C, 4 °C, and 3.5 °C; in 40% state—accordingly, 6.5 °C, 5 °C, and 4.5 °C; and in 50% state the difference is 8 °C EFH1 and 6 °C for EFH2 and EFH3. The obtained results show that the target temperature may be not reached when using compression support in the stretched state required to generate some particular compression. The decrease of temperature depending on the sample stretch can be explained by the increase in surface area during stretching, while the amount of electro-conductive yarn in the knitted specimen remains the same. A complete knit loop is a combined form of a needle loop, which is composed of a head and two side limbs and a sinker loop. The tensile force counteracts the friction force that occurs at the contact point between the loops during the stretch of conductive fabric, and contributes to the displacement of the contact point. Additionally, the yarn’s bending curvature is altered. Furthermore, due to the movement of the contact points, the length of the limbs, head, and sinker loop changes. The degree of the movement of the contact points is reduced as the tensile force further increases, and the contact pressure between the head and sinker loop rises rapidly. By superposition of the length-related and contact resistance of the fabric, the resistance of the conductive knitted fabric along the course direction could be clarified. During the initial stretching process, the contact resistance acts as a decreasing factor, while the length-related resistance dominates the total equivalent resistance in the stretching process for the further increase of the tensile force. The heat, lost by radiation and air convection, is higher during stretching as the area of designed fabrics increases. Finally, the saturation of heat is achieved between the heat generation and the heat loss in a lower level compared to the normal state of fabrics [29]. Thus, the stretch effect on heat generation has to be considered during the development of heated compression supports.

### 3.3. Effect of Washing on Structural and Temperature (T) Characteristics

During the washing process, various stresses may damage the conductive silver coating from electrically conductive yarns [30]. In order to investigate the influence of washing on heating changes, the specimens were washed five times and structural and temperature changes after the first, second, third, and fifth washing and drying cycles were measured.

**Figure 7:**
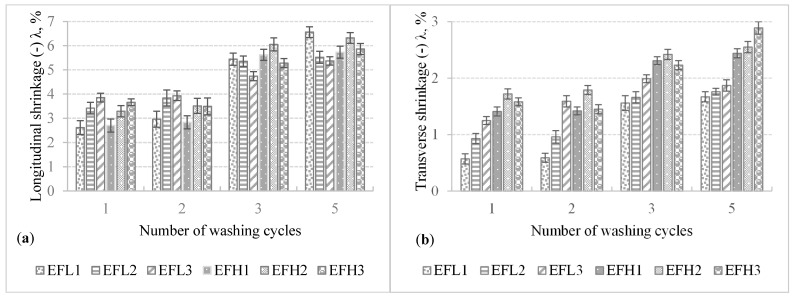
Shrinkage in the longitudinal (**a**) and transverse direction (**b**) of the developed electro-conductive fabrics upon the number of washing.

The shrinkage during laundering is a major concerning point for weft knitted fabrics [31]. Knitted fabrics along with elastomeric yarns are stretchable structures that may change their dimension while exposed to wet processes. The cumulative impact of various factors such as relaxing, finishing, drying, and machinery effects are responsible for shrinkage (a sign “−” symbolizes the sample shrunk, and “+”—the sample lengthened). All parameters during washing and drying were kept constant for each cycle. Dimensional changes in longitudinal (wale) and transverse (course) directions after washing and drying of the investigated fabrics are presented in Figure 7.

As it can be seen in Figure 6, all investigated specimens shrunk after washing and drying. Shrinkage values in the longitudinal direction are significantly (2–5 times) higher than in the transverse direction due to the thick (114.5 tex) elastomeric inlay yarn laid in the course direction. The shrinkage of less than ±5% is considered insignificant. As it can be seen in Figure 6, the transverse shrinkage of all fabrics after the first and even fifth washing cycles does not exceed −3%. The longitudinal shrinkage after the first and second washing cycles does not exceed −4%, but after the fifth washing and drying cycle, it reached −(5.4–6.6)%. The shrinkage is not very high; however, it may influence the surface resistivity due to the decreased surface area of the sample. Instead of yarn or loop length shrink, the dimensional changes occurring after laundering were primarily due to changes in the loop shape. After each washing cycle, the fabrics had taken up their absolutely relaxed dimensions, there were no major changes that occurred in yarn linear density.

**Figure 8:**
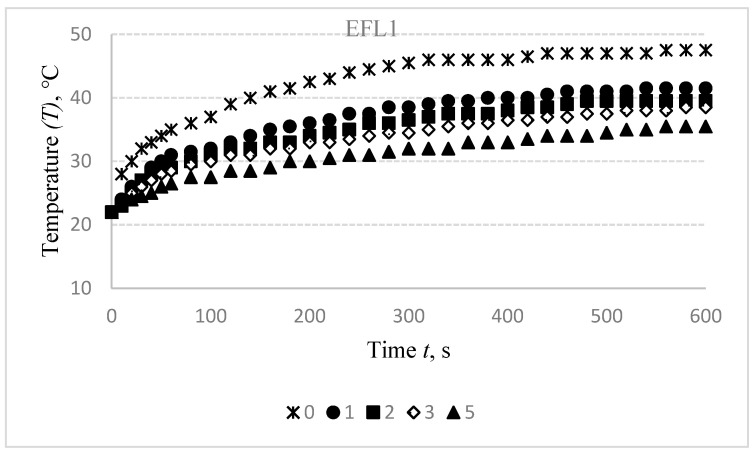

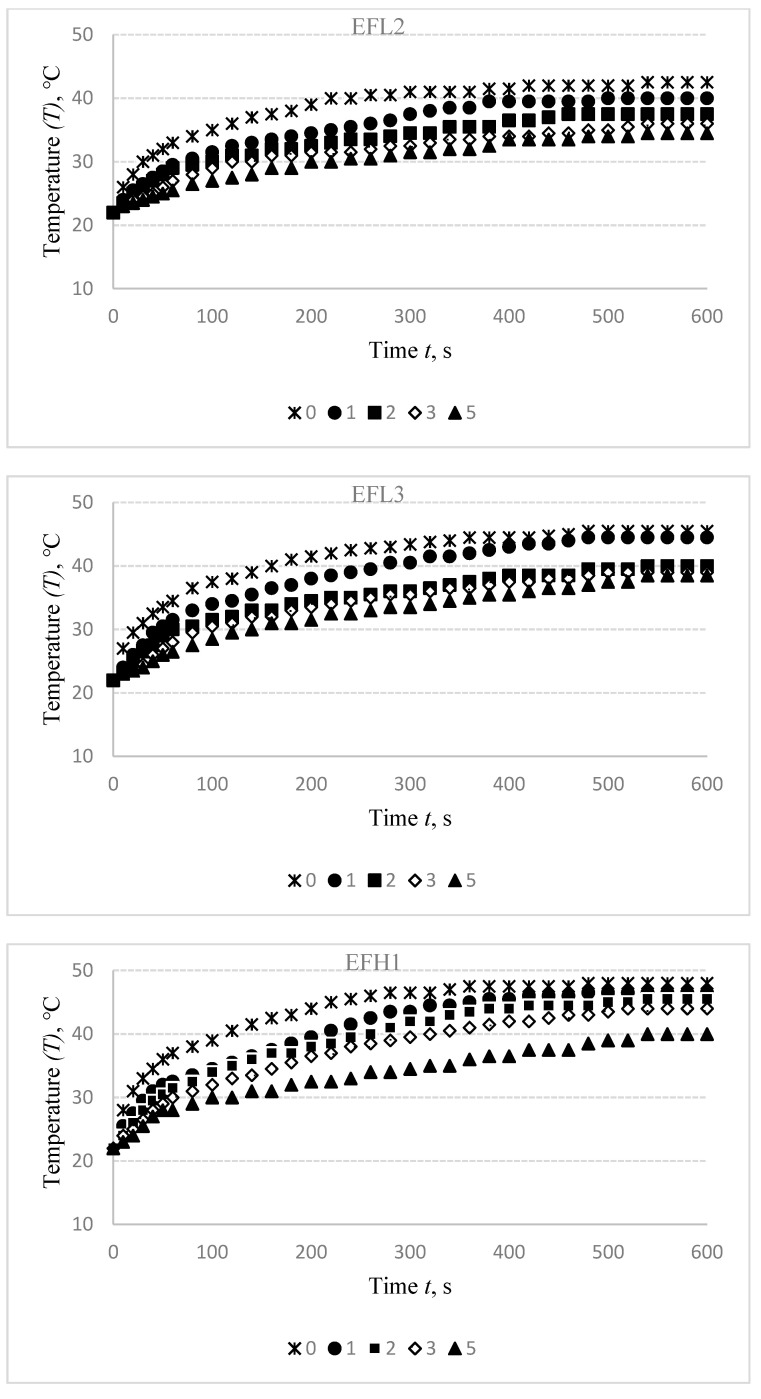

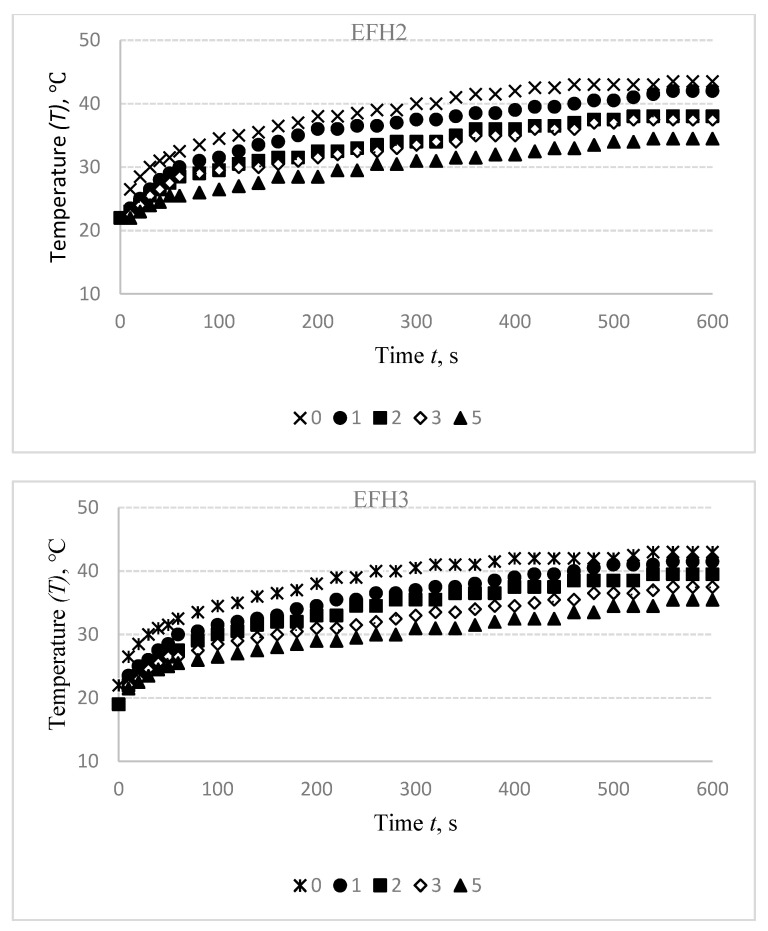
Temperature changes of EFL1, EFL2, EFL3, EFH1, EFH2, and EFH3 structured electro-conductive fabric during the 600 s period before washing and after different wash cycles: 1st, 2nd, 3rd, 5th.

Results of the temperature changes after washing off the electro-conductive specimens in the initial, i.e., non-stretched state are presented in Figure 8.

Washing of the compressive electro-conductive knitted fabric has a detrimental influence on heat generation, as shown in Figure 8. It was found that the difference between the temperature of non-washed specimen and the first time washed after 600 s is 6 °C for EFL1, 2.5 °C for EFL2, and 1 °C for EFL3, while this difference after the second washing cycle is, accordingly, 8 °C, 5 °C, and 4.5 °C; after the third washing cycle—9 °C, 6.5 °C, and 6.5 °C; and after the fifth washing cycle is, accordingly, 12 °C, 8 °C, and 7 °C. For EFH group structures, this difference between temperatures of the non-washed and the first-time washed fabrics is 1 °C for EFH1, 0.5 °C for EFH2, and 1.5 °C for EFH3; after the second washing cycle it is, accordingly, 2.5 °C, 4 °C, and 3.5 °C; after the third washing cycle—accordingly, 4 °C, 6.5 °C, and 5.5 °C; and after the fifth washing cycle the difference is, accordingly, 6 °C, 9 °C, and 6.5 °C. This phenomenon can be explained by the removal of the conductive coating from the yarn.

**Figure 9:**
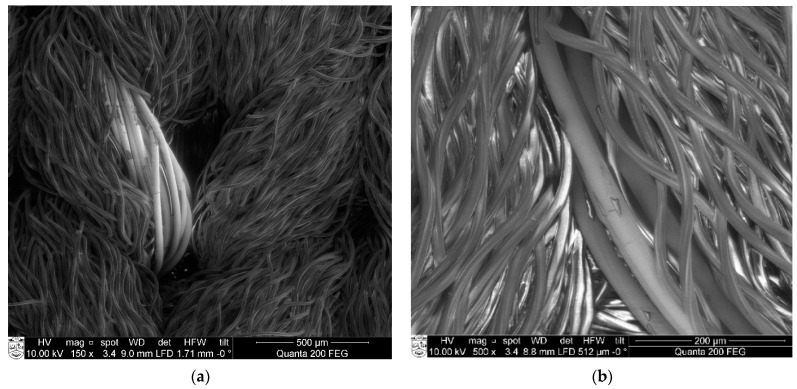
SEM images of specimens knitted with electro-conductive yarn: (**a**) 150× and (**b**) 500× magnification before wash.

The experimental results showed that despite the chosen plated structure, the protection efficiency against damaging impact during laundering is not sufficient enough. In fact, water with detergent penetrates by diffusion or other capillary processes mechanical protections (realized by plating with non-conductive yarns), and after penetration, it damages the silver coating of the conductive yarns. Due to the removal of silver particles, the flow of electricity was interrupted, and resistance got higher. Silver coated polyamide yarns can be and often are protected within e-textile structures by bridging simple non-conductive yarns over them. Nevertheless, this protection is effective against mechanical impacts (abrasion) during laundering, but it does not protect conductive yarns against water and chemical impact, especially after several washing cycles [32]. In Figure 9, SEM images of the knitted structure of the electro-conductive compression specimen are presented, which demonstrate that electro-conductive yarn, laid in the plated structure as the ground yarn, in some places goes up into the fabric surface (this could happen because of uneven yarn tension during the knitting of plated structure) and may be mechanically damaged. Therefore, to ensure conductivity in the entire length of the yarn, multifilament yarns are used. Electro-conductive yarns or at least individual filaments of the yarn, being in the inner structure of the knit and covered by the plating yarn, are protected from the main mechanical impacts, such as abrasion.

**Figure 10:**
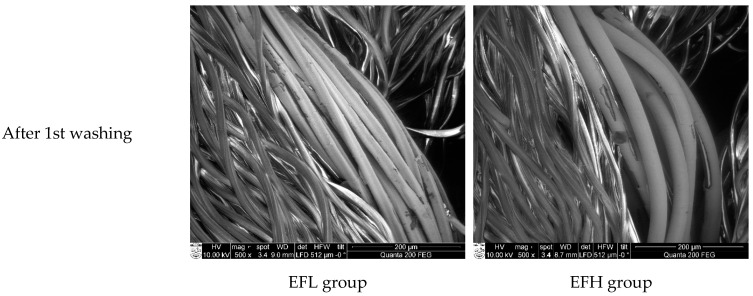

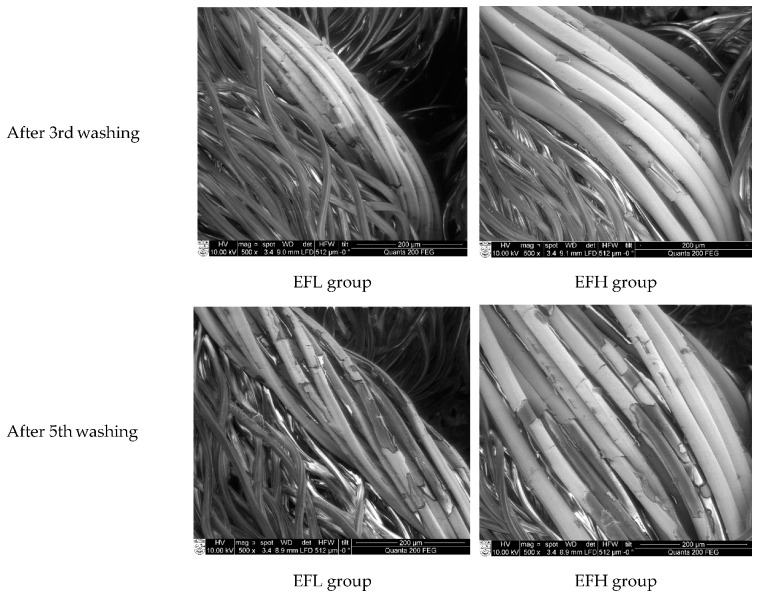
SEM images of electro-conductive yarns in EFL and EFH group fabrics after 1st, 3rd, and 5th washing cycles (500× magnification).

SEM images of EFL and EFH specimens after different numbers of washing and drying cycles are shown in Figure 10 and demonstrate the increasingly growing area of damages on the surface of the conductive coating.

Immersion in liquids causes the yarn to experience some surface damages. This damage affects the silver-plated layer of the yarn and eventually affects the conductivity of the yarn present in the fabric structure if the silver coating is damaged. Removal of silver directly affects the resistance, and it increases because the number of charge-carrying particles decreases. Consequently, the temperature generated on the fabric surface is lower than before washing. However, the removal of silver coating from the particular points of the filaments in the yarn is not in an entire length and this ensures continuous conductivity. The electrical resistance of the knitted fabric is collectively contributed by the inter-connection of the conductive yarns within the fabric [33].

**Figure 11:**
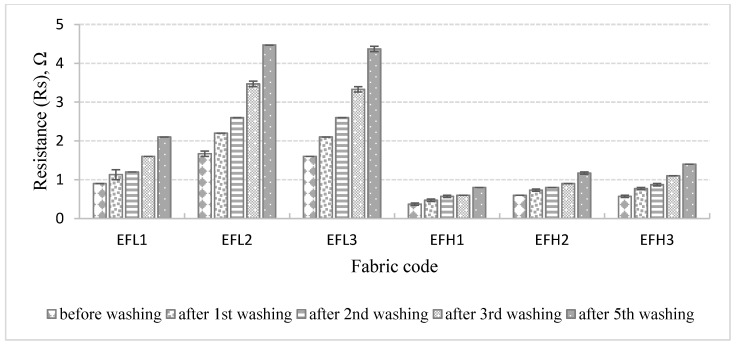
Resistance of EFL1, EFL2, EFL3, EFH1, EFH2, and EFH3 structured electro-conductive fabrics before and after different numbers of washing and drying cycles.

Resistance values of all specimens fabricated with different layouts of the electro-conductive yarn in the knitting pattern before and after washing were measured and are presented in Figure 11.

As it can be seen from the presented results, the resistance increases after each washing cycle as the quantity of conductive particles, their mutual distance, and the area of contact between the particles has been changed during washing. The resistance of the knitted fabrics is very sensitive to the quantity of conductive yarns in the structure and the quantity of the conductive particles on the yarns. Resistance rises gradually with the decrease of the area of the conductive surface on the electro-conductive yarn in the knitting pattern. The obtained outcomes denote that the knitting structure, linear density of conductive yarn used, and the number of washing cycles have an influence on the fabric resistance. The EFH group of knits demonstrates lower resistance in comparison with the EFL group because of the higher linear density of the electro-conductive yarn and a higher number of filaments in the yarn. Consequently, changes in the resistance of EFH group fabrics after washing are much less in comparison with EFL group fabrics.

**Figure 12:**
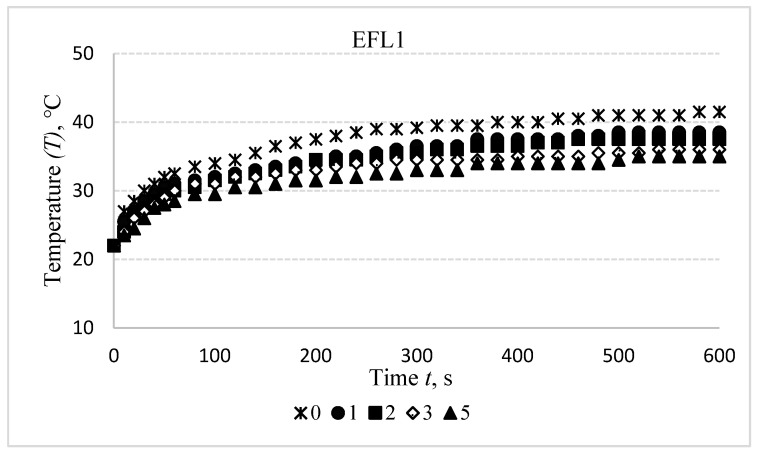

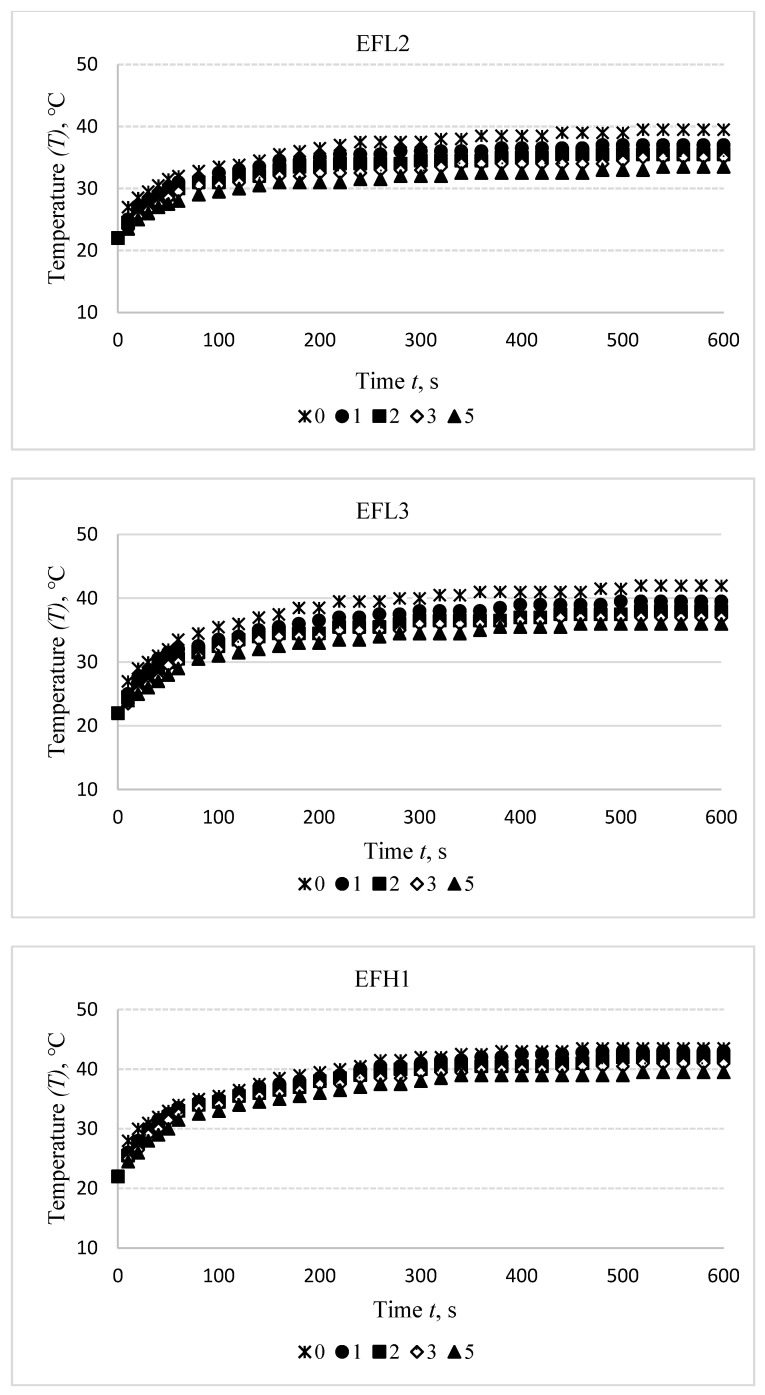

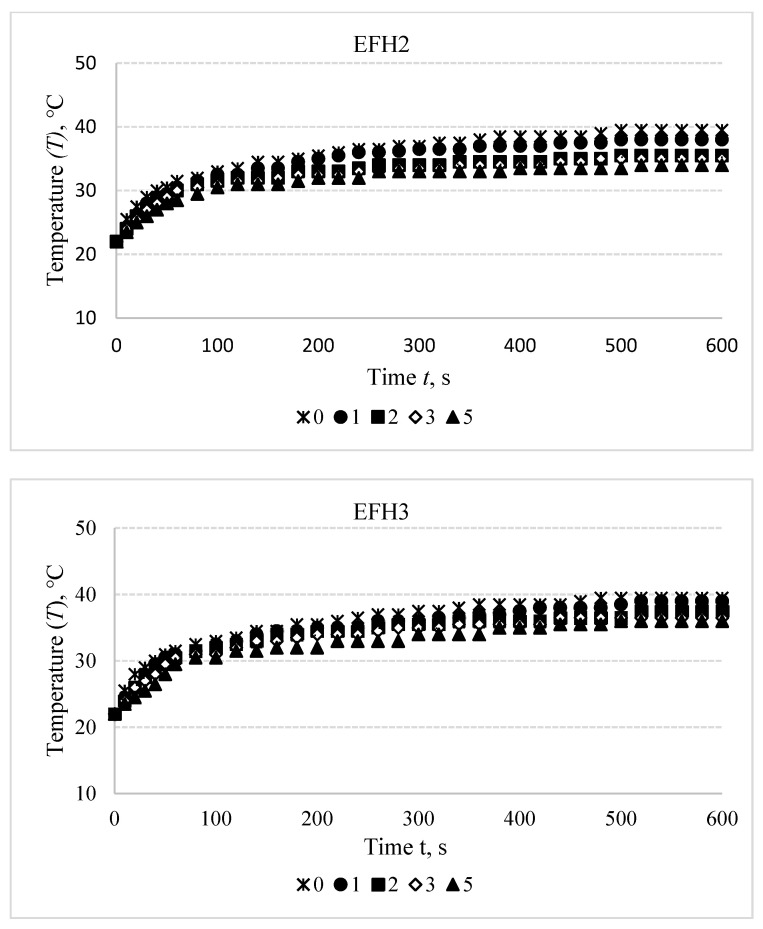
Temperature changes of EFL1, EFL2, EFL3, EFH1, EFH2, and EFH3 structured electro-conductive fabric during the 600 s period before and after different washing cycles (1, 2, 3, 5) at 30% stretch.

To uncover the control of the stretching effect on the temperature generation after washing, the washed specimens were stretched to 30%. The obtained results are presented in Figure 12.

As shown by the results in Figure 8 and Figure 12, the heat generation dynamics during the time have a similar character in both non-stretched and stretched states. However, at the 30% stretched state, the influence of washing on the decrease of temperature is approximately 1 °C lower in comparison with the same specimens in a non-stretched state. Anyway, the washed specimens did not reach the 40 °C temperature even after 10 min, while non-washed and non-stretched fabrics reached this temperature in approximately 2–3 min. Thus, the possible heating changes due to the stretching and washing impacts must be taken into consideration during the designing phase of the heated compression knits.

## 4. Conclusions

The presented work revealed possibilities to use electrically conductive textile for heat generation in orthopaedic compression supports. Silver coated polyamide yarns were knitted following a combined half-Milano rib structure plated by the PA6.6 yarn to secure it from mechanical abrasion. The temperature of the developed samples rises rapidly for the first minute, then slows down afterward. The relatively stable 40 °C heating temperature was reached in approximately 2–3 min depending on specimen structure and voltage applied. The higher linear density of the conductive yarn requires a lower voltage to reach the required temperature.

It was obtained that due to the increase of the surface area, the stretch of the specimen has a negative impact on heat generation. After 600 s heating period, 50% stretch resulted in approximately 8 °C decrease in the final temperature of the specimens with the highest density of the electro-conductive yarns in the structure (EFL1 and EFH1), while for fabrics with the twice lower amount of electro-conductive yarns in the structure (EFL2, EFH2, and EFL3, EFH3) this decrease was 4–6 °C. The same tendency was observed at all stretch levels.

A negative effect of washing on heat generation was observed, too. Removal of silver coatings during the washing directly affects the resistance. It increases and, consequently, the temperature generated on the fabric surface is lower than before washing. However, a partial removal of silver coating from the yarn retained the electrical conductivity. The electrical resistance of the knitted fabric is collectively contributed by the inter-connection of the conductive yarns within the fabric. The decrease of temperature of fabrics with the highest density of electro-conductive yarn in the knitting structure (EFL1 and EFH1) was significantly higher after each washing cycle in comparison with the fabrics with the twice lower amount of electro-conductive yarns in the structure (EFL2, EFH2, and EFL3, EFH3). However, at the 30% stretched state, the influence of washing on the decrease of temperature is approximately 1 °C lower in comparison with the temperature of the fabrics in a non-stretched state. Thus, the possible heating changes due to the stretching and washing impacts must be taken into account during the designing phase of the heated compression knits.

In the next research phase, the authors will investigate the possibilities of heating temperature regulation due to structural and morphological changes under stretching and washing impact to improve the performance of the knit compression garments for orthopaedic applications.

## Figures and Tables

**Table 1:** Knitting structures and distribution of electro-conductive yarn in the knitting pattern.

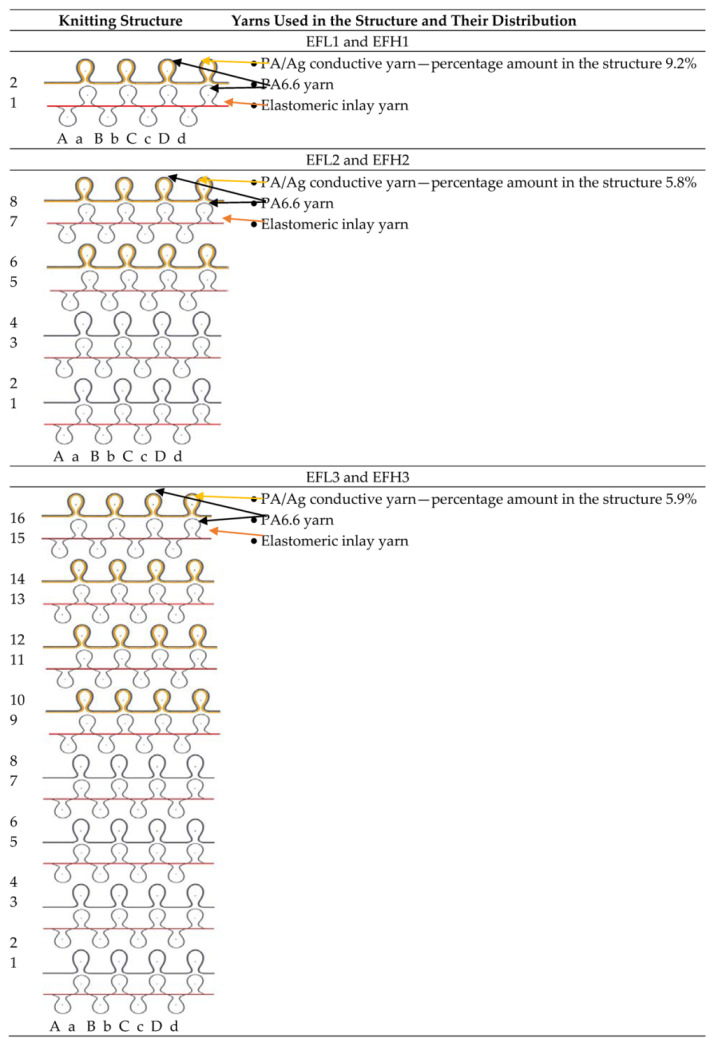

Note: EFL—linear density of electro-conductive yarn used in knitted structure—66 tex (12 filaments); EFH—linear density of electro-conductive yarn used in knitted structure—235 tex (34 filaments).

**Table 2:** Main characteristics of designed samples.

Sample Code	Area Density, g/m^2^	Wale *P_w_* and Course *P_c_* Density (cm^−1^)				Average Loop Length, (mm)	Sample Area, m^2^
		Technical Face Side		Technical Back Side			
		*P_c_*	*P_w_*	*P_c_*	*P_w_*		
EFL1	326 ± 2	6.0 ± 0.1	6.0 ± 0.1	12.0 ± 0.1	6.0 ± 0.1	8.2 ± 0.3	0.022
EFL2	324 ± 2	6.0 ± 0.1	6.0 ± 0.1	12.0 ± 0.1	6.0 ± 0.1	8.2 ± 0.2	0.022
EFL3	325 ± 2	6.0 ± 0.1	6.0 ± 0.1	12.0 ± 0.1	6.0 ± 0.1	8.2 ± 0.3	0.022
EFH1	351 ± 2	6.0 ± 0.1	6.0 ± 0.1	12.0 ± 0.1	6.0 ± 0.1	8.1 ± 0.2	0.022
EFH2	349 ± 2	6.0 ± 0.1	6.0 ± 0.1	12.0 ± 0.1	6.0 ± 0.1	8.1 ± 0.2	0.022
EFH3	350 ± 2	6.0 ± 0.1	6.0 ± 0.1	12.0 ± 0.1	6.0 ± 0.1	8.1 ± 0.3	0.022

**Table 3:** Tensile force and corresponding compression at the different stretch levels.

Sample Code	Tensile Force *F*, N					Compression *P*, kPa				
	10%	20%	30%	40%	50%	10%	20%	30%	40%	50%
EFL1	6.88 ± 0	10.32 ± 0.31	14.45 ± 0.65	18.64 ± 1.33	23.01 ± 1.92	3.07	4.59	6.44	8.30	10.25
EFL2	7.59 ± 0.06	11.28 ± 0.16	14.86 ± 0.07	18.49 ± 0.11	22.61 ± 0.38	3.38	5.02	6.62	8.24	10.07
EFL3	7.89 ± 0.13	11.44 ± 0.08	15.44 ± 0.14	19.64 ± 0.72	24.06 ± 0.66	3.52	5.09	6.88	8.75	10.72
EFH1	8.1 ± 0.04	12.92 ± 0.14	19.13 ± 0.77	25.04 ± 0.39	29.24 ± 0.81	3.61	5.76	8.52	11.16	13.03
EFH2	8.29 ± 0.08	12.07 ± 0.05	16.24 ± 0.2	20.68 ± 0.79	24.84 ± 0.56	3.69	5.38	7.24	9.22	11.07
EFH3	8.25 ± 0.13	12.19 ± 0.14	16.51 ± 0.1	21.03 ± 0.19	25.92 ± 0.23	3.68	5.43	7.36	9.37	11.55

## Data Availability

The data presented in this study are available on request from the corresponding author.
